# Genomic and phenotypic characterization of *Klebsiella pneumoniae* phage KP Ø1: a novel lytic *Slopekvirus* targeting uropathogenic multidrug-resistant *Klebsiella pneumoniae*

**DOI:** 10.1038/s41598-026-67695-4

**Published:** 2026-09-03

**Authors:** Reham Fadl, Ghada Ismail, Ahmed Elshafei, Amira Mohamed Mokhtar, Omnia Mohamed El Nabawy, Mohamed Gomaa Seadawy, Mahmoud M. Baraka, Basma Sherif

**Affiliations:** 1 Clinical Pathology Department, Faculty of Medicine, Ain Shams University, Cairo, Egypt; 2 Urology Department, Faculty of Medicine, Modern University for Information and Technology, Cairo, Egypt; 3 Biodefense Center for Infectious and Emerging Diseases, Ministry of Defense, MOD, Cairo, Egypt

**Keywords:** *Klebsiella pneumoniae*, Phage therapy, Genomic characterization, *Slopekvirus*, MDR uropathogens, Microbiology, Molecular biology

## Abstract

The rise of multidrug-resistant (MDR) uropathogenic gram-negative bacteria (GNB) necessitates the development of alternative therapeutic strategies. This study aimed to isolate, phenotypically characterize, and perform whole-genome sequencing of the bacteriophage demonstrating the broadest host range against MDR uropathogens. Fifty MDR GNB isolates were screened for lytic phages. The most promising candidate, *Klebsiella pneumoniae* phage KP Ø1, was characterized using plaque assay, Transmission Electron Microscopy (TEM), and pH/thermal stability testing. Genomic characterization was performed via whole-genome sequencing (WGS), with functional annotation and lifestyle prediction using PhaBOX and PhageScope software. *Klebsiella pneumoniae* was the most prevalent MDR uropathogen. *Klebsiella pneumoniae* phage KP Ø1 exhibited a 50% host range and high lytic titer (10⁸ PFU/mL). TEM revealed an icosahedral head and short contractile tail. Genomic characterization by WGS revealed that *Klebsiella pneumoniae* phage KP Ø1 possesses a 174,591 bp double-stranded deoxyribonucleic acid (dsDNA) genome containing 274 predicted open reading frames (ORFs). No lysogeny-related genes, toxins, or antibiotic resistance markers were detected, confirming its strictly lytic nature and supporting its potential as a candidate for phage therapy applications. The phage remained stable (10⁸ PFU/mL) across temperatures of − 20 °C to 50 °C; supporting its suitability for long-term biobanking and suggesting potential activity at physiological temperature, and across a pH range of 7–9. *Klebsiella pneumoniae* phage KP Ø1 is a novel, obligately lytic *Slopekvirus* whose genomic architecture, stability profile, and absence of lysogeny-associated, virulence, and antimicrobial resistance genes ( AMR) collectively support its candidacy for further preclinical evaluation as a phage therapy agent against uropathogenic MDR *Klebsiella pneumoniae*.

**Supplementary Information:**

The online version contains supplementary material available at https://doi.org/10.1038/s41598-026-67695-4.

## Introduction

Urinary tract infections (UTIs) are major public health challenge that affects quality of life and also imposes a substantial social burden due to medical expenses^1^. Owing to their high recurrence and chronicity, they are the second most common bacterial infections after pneumonia. UTIs are also a prominent cause of septicemia^2^. Uropathogens have specific characteristics that help them colonize the urinary tract; these include producing adhesins that aid invasion of the bladder and renal epithelium and producing toxins, proteases, and siderophores, that allow the pathogen to acquire nutrients from host cells^1^. Biofilm formation is another important characteristic that makes uropathogens more resistant to antibiotics and the host immune response. Antimicrobial abuse also drives the rapid development of MDR uropathogens^3^. Multi-drug resistant bacteria are those resistant to at least one agent in three or more antimicrobial groups^4^.

The widespread occurrence of MDR strains has a serious impact on human morbidity and mortality^5^. Accordingly, several studies have suggested phage therapy as a potential therapeutic alternative against MDR microorganisms because bacterial resistance mechanisms to phages differ from those to antibiotics. *Escherichia coli (E. coli)*,* Klebsiella pneumoniae (K. pneumoniae)*,* and Proteus mirabilis (P. mirabilis)* are the most common uropathogenic bacteria for which phage therapy has been applied^6^.

Interestingly, phages can prevent biofilm formation by inducing or producing polysaccharide depolymerase, providing an effective solution against catheter-associated UTI^7^. Phages can persist in the human body for several days after a single oral dose. Additionally, when bacteria are killed, phage titer decreases until phages are completely removed from the body through excretion. Compared with antimicrobial therapy, phage therapy showed no additional adverse effects. Furthermore, phages did not affect the normal microbiota and did not induce dysbiosis. Thus, bacteriophages can be an alternative therapeutic tool to selectively destroy pathogenic agents and help address the drug resistance obstacle among bacteria^8^.

The aim of the present study was to isolate, phenotypically characterize, and perform whole-genome sequencing of the bacteriophage demonstrating the broadest host range against MDR uropathogens.

## Materials and methods

A cross-sectional study was conducted on 50 MDR-GN uropathogens at Reference Laboratory for Egypt University Hospitals from the Main Microbiology Laboratory, Clinical Pathology Department, Ain Shams University Hospitals, from September 2021 to February 2025. All experiments were performed in accordance with relevant guidelines and regulations. The need to obtain informed consent was waived by the Ethical Research Committee, Faculty of Medicine, Ain Shams University, on 17/09/2022, No. FMASU MD246/2022, because the study used bacterial isolates with no direct patient involvement or identifiable patient information.

Phenotypic bacterial identification and antimicrobial susceptibility testing (AST) were performed using the VITEK 2-Compact System (Biomérieux, France) according to the Clinical and Laboratory Standards Institute (CLSI) 2021 guidelines. Bacterial isolates were preserved in tryptic soy broth (TSB) (Oxoid, UK) at − 80 °C for further testing.

### Preparation of host strain inoculum culture for phage analysis

One vial of stock culture was thawed at room temperature (RT) and streaked onto a MacConkey agar plate, then incubated for 24 h (h) at 36 ± 1 °C. 2–3 colonies were inoculated into 10 mL prewarmed nutrient broth, and incubated until the mid-log phase, indicated by turbidity^9^.

### Isolation, purification, and propagation of phages from sewage water

Sewage sample was added to 50 mL Falcon tubes, centrifuged (6000 g, 20 min), and the supernatant was recovered into a new sterile Falcon tube. 100 µL of bacterial suspension was added to 10 mL double-strength nutrient broth (2 x NB), and 10 mL of filtered supernatant was added to a sterile beaker. The mixture was incubated at 36 ± 1 °C for 24 h under agitation (120–200 rpm). The enriched sample was then poured into 50 mL Falcon tubes and centrifuged (6000 g for 20 min). The supernatant (phage lysate) was filtered through a 0.45 μm pore-sized polyvinylidene fluoride (PVDF) filter to remove bacterial cells and cellular debris. The phage lysate was aseptically collected and stored at 4 °C^10^.

Qualitative screening for bacteriophages qualitatively by spot test 100 µL of bacterial suspension was added to 3 mL of melted soft agar, mixed well, and poured onto nutrient agar plates. Once the overlay agar layer had completely solidified, each plate was divided into four quadrants. 10–20 µl of each lysate was spotted; and left to dry. Plates were incubated overnight at 36 ± 1 °C, then examined for clear and turbid lysis plaques indicative of phage presence. The next day, the phage lysate with consistently positive spot test results was processed for the quantitative plaque assay^11^.

Quantitative plaque assay was performed using the standard double agar overlay (DAO) technique, with phage lysate serially diluted 10-fold (10^− 9^) in SM buffer (Himedia, India)^12^. A row of 9 sterile Eppendorf tubes was set, and aseptically, 900 µL SM buffer was added to each. 100 µL of phage lysate was added to the first Eppendorf, mixed, and 100 µL was then transferred to the next Eppendorf in the series. 10-fold dilutions were prepared. In the last tube, the phage preparation would have been diluted to 10^− 9^. From each dilution, 0.5 mL was added to 0.5 mL of host culture and 3 mL of melted soft agar, vortexed well, and poured onto top of the underlay agar. The overlay was allowed to solidify completely at RT before the plates were inverted and incubated at 36 ± 1 °C for 24 h. The following day, plaques were examined and counted using the following equation: Phage titer in plaque forming unit per ml (PFU /ml) = number of plaques/ (volume of lysate infected × dilution factor)^13^. A single plaque was picked from the agar layer using a sterile micropipette tip, suspended in 300 µL nutrient broth with 3 µL bacterial host, vortexed for 10 s, and then left for 4 h at 4 °C. Each phage lysate was centrifuged at 6000 g for 20 min, and the supernatant was collected aseptically, and kept at 4 °C for use in a serial dilution test for further purification. This purification procedure was repeated seven times for each isolated bacteriophage to produce purified phage stocks. One ml of purified phage lysate obtained on the 7th time was added to 9 mL of mid-log phase bacterial growth in nutrient broth, then incubated for 4–6 h in a shaking incubator at 36 ± 1 °C, and serial dilution was repeated as mentioned in 3.3. This step was repeated until a high titer was reached, and the phage stock volume was then stored at 4 °C^14^.

### Host range determination

The isolated phages (thirteen phages) were tested against fifty bacterial isolates using a spot test. A 3 mL volume of soft agar (0.7% agar) medium was inoculated with 100 µL of the bacterial isolate and poured onto nutrient agar plates. Plates were allowed to solidify, and 10 µL of each purified phage lysate was spotted twice onto the surface of the plates. Plates were then incubated at 36 ± 1 °C for 24 h. After overnight incubation, plates were examined for clear spots on the bacterial lawn, indicating bacterial lysis^15^.

### Evaluation of characteristics of the broadest host range bacteriophage

*K. pneumoniae* KP Ø1.

#### Morphology by transmission electron microscopy (TEM) 

A high-titer stock of *K. pneumoniae* phage KP Ø1 (10^8^ PFU /mL) was centrifuged at 12,000 g for 60 min. The supernatant was filtered through 0.45 μm pore-sized polyvinylidene fluoride (PVDF) filters, then placed on carbon grids (200 mesh) coated with formvar and left for 2 min. The phage was stained with 2% uranyl acetate (negative stain), and excess dye was removed with filter paper^10^. Sample examination was conducted at the Regional Center of Mycology and Biotechnology, Al-Azhar University, Cairo, Egypt, using electron microscopy (Beckman 1010, operated at 80 kV).

#### Physical properties of *K. pneumoniae* phage KP Ø1

##### Temperature stability

200 µl of *K. pneumoniae* phage KP Ø1 (10^8^ PFU /ml) lysate was assessed for stability at different temperatures (-20 °C, 4 °C, 37 °C, 50 °C, 60 °C, 70 °C, and 80 °C) after a 1 h incubation in a water bath to confirm suitability for long-term biobanking and in vivo application at physiological temperature. Serial dilutions of KP Ø1 were prepared as described in 3.3 and spotted twice immediately after incubation using the double-layer standard technique on a lawn of the bacterial host strain to estimate phage titers^16^.

##### pH stability

100 µl of *K. pneumoniae* phage KP Ø1 (10^8^ PFU /ml) was added to 900 µl of buffer at different pH levels (1, 2, 4, 5, 7, 9, 10, 12, 13) and stability was assessed after 1 h. Serial dilutions of phage were prepared as described in 3.3 and spotted twice using the double-layer standard technique on a lawn of the bacterial host strain to estimate phage titers^16^. Different pH levels were adjusted by adding 1 N hydrochloric acid (1 N Hcl) and 1 N sodium hydroxide (1 N NaOH) to distilled water (DW) to reach the desired pH.

### Whole genome sequencing of *K. pneumoniae* phage KP Ø1

#### Nucleic acid extraction of *K. pneumoniae* phage KP Ø1

Nucleic acid was extracted from highly purified high-titer (10^10^ PFU/mL) lysates using a modified proteinase K/ Sodium Dodecyl Sulfate (proteinase K/SDS) method^17^. The purified phage (600 µL) was treated with DNase I and RNase A (1 mg/L) at 37 °C overnight, followed by enzyme inactivation at 80 °C for 15 min. Ethylenediaminetetraacetic acid (EDTA) (0.02 mol/L), proteinase K (50 mg/L), and Sodium Dodecyl Sulfate (SDS) (0.5%) were added, and the mixture was incubated at 56 °C for 1 h. Phenol extraction was performed twice: first with balanced phenol (650 µL) and then with phenol/chloroform/isoamyl alcohol (25:24:1). The final aqueous phase was mixed with isoamyl alcohol (550 µL) and incubated at − 20 °C for 2 h. After centrifugation (12,000 g, 4 °C, 15 min), the pellet was washed with 75% ethanol, dried, resuspended in nuclease-free water, and stored at − 20 °C. The quantity of nucleic acid was measured using a by Qubit 2.0 Fluorometer (Thermofisher scientific, USA).

#### Sequencing of extracted and purified product

Purified products were sequenced using an Illumina MiSeq platform (Illumina, USA), generating paired-end reads with a mean read depth of 50× coverage. Sequencing library was constructed using the NEBNext^®^ Ultra™ II DNA Library Prep Kit (New England Biolabs, USA).

#### Genomic assembly and annotation

Raw sequencing data for *K. pneumoniae* phage KP Ø1 were assembled using Newbler v3.0, and genome annotation was performed with the Rapid Annotation using Subsystem Technology (RAST) online tool. Another round of annotation was performed after RAST annotation to confirm assigned functions or to assign functions to proteins with unassigned functions using (BLASTp) against the National Center of Biotechnology Information (NCBI) database, PhaBOX^18^, and PhageScope^19^ online platforms. Genome annotation confidence was assessed using PhageScope HMM-based taxonomic annotation, achieving a 100% hit rate across 262 HMM hits. Genome completeness and quality were assessed using CheckV v0.8.1 implemented within the PhageScope annotation pipeline. Assembly metrics, including genome completeness and contamination score, were evaluated. Phage termini were identified using a computational method that analyzes NGS data, specifically focusing on read alignment files^20^.

Comprehensive genomic safety screening was performed using PhageScope (Task ID: 41733), including: virulence factor detection against the Virulence Factor Database (VFDB), antimicrobial resistance gene (AMR) screening against the Comprehensive Antibiotic Resistance Database (CARD), anti-CRISPR protein annotation, and CRISPR array detection within the phage genome to evaluate the phage’s potential for therapeutic applications.

Computational host assignment was performed using two independent methods: PhageScope Host Assignment module employing DeepHost and BLAST-based analysis with TaskID: 41733, and PhaBOX CHERRY module using amino acid identity (AAI)-based prediction with JobID: PhaBOX-20,260,624-BRZQEXJZYG. The whole genome sequence data of *K. pneumoniae* phage KP Ø1 was submitted to the NCBI Genebank database with accession number: PZ022450.

#### Phylogenetic analysis of *K. pneumoniae* phage KP Ø1

The complete genome sequence of *K. pneumoniae* phage KP Ø1 was compared against available bacteriophage genome sequences deposited in the NCBI GenBank database using BLASTn (Basic Local Alignment Search Tool for nucleotides; https://blast.ncbi.nlm.nih.gov/) with an E-value threshold of 1 × 10⁻⁵ to identify closely related phages. The top hits sharing significant nucleotide similarity with *K. pneumoniae* phage KP Ø1 were selected for phylogenomic analysis, with priority given to phages possessing complete genome sequences and representatives spanning diverse Enterobacteriaceae infecting phage genera to provide adequate phylogenetic context. Genome sequences of all selected reference phages were retrieved in FASTA format from the NCBI GenBank database.

Multiple sequence alignment of the complete genome sequences of *K. pneumoniae* phage KP Ø1 and all reference phages was performed using the MUSCLE algorithm (Multiple Sequence Comparison by Log-Expectation) integrated within MEGA12 (Molecular Evolutionary Genetics Analysis version 12; https://www.megasoftware.net/) with default parameters. The aligned sequences were manually inspected and trimmed to remove poorly conserved terminal regions prior to phylogenetic tree construction. Statistical robustness of all internal nodes was evaluated by non-parametric bootstrap analysis with 1,000 replicates; bootstrap support values are displayed as percentages at each node, and nodes with support values below 50% were not shown. The final phylogenomic tree was visualized and annotated using the built-in tree explorer in MEGA12, and taxonomic classification of *K. pneumoniae* phage KP Ø1 was interpreted in accordance with the current guidelines of the International Committee on Taxonomy of Viruses (ICTV), with genus and family level assignments informed by the observed clustering patterns in conjunction with genomic features including genome size, GC content, and the presence of conserved hallmark genes characteristic of the genus *Slopekvirus*, family *Straboviridae*.

## Results

### Bacterial identification and antimicrobial susceptibility profile

The study was conducted on 50 MDR-GN uropathogens: *K. pneumoniae*, 43 (86%), and *E. coli*, 7 (14%) isolates. All isolates were identified and were resistant to at least one agent in three different antimicrobial groups by VITEK 2- Compact System (Biomérieux, France). *K.pneumoniae* isolates were resistant (100%) to piperacillin/tazobactam (TZP), ampicillin/sulbactam (SAM), ceftazidime (CAZ), ceftriaxone (CRO), cefoxitin (FOX), cefepime (FEP), cefazolin (CFZ), meropenem (MEM), ciprofloxacin (CIP), nitrofurantoin (F) and trimethoprim/sulfamethoxazole (SXT), while *E.coli* isolates were resistant (100%) to ampicillin (AMP), ampicillin/sulbactam (SAM), ceftazidime (CAZ), ceftriaxone (CRO), cefoxitin (FOX), cefepime (FEP), cefazolin (CFZ), tobramycin (TOB), ciprofloxacin (CIP), levofloxacin (LEV) and trimethoprim/sulfamethoxazole (SXT). *K.pneumoniae* isolates were resistant to amikacin, gentamicin, tobramycin, and levofloxacin (88.4%, 60%, 98%, 98%, respectively), while *E.coli* isolates were resistant to piperacillin/tazobactam, meropenem, amikacin, gentamicin, and nitrofurantoin (86%, 86%, 29%, 71%, 29%, respectively). Antimicrobial resistance patterns are shown according to clinical & laboratory standards institute 2021(CLSI) classes in Supplementary Table S1.

### Phage isolation and host range determination

Ten bacteriophages specific to *E. coli* and three specific to *K. pneumoniae* were isolated from sewage water collected from different places. The infectivity of 13 phages was assessed against all (50) MDR GN bacterial isolates to determine the host range of each phage and was shown in Supplementary Table S2.

### Morphological characterization of *K. pneumoniae* phage KP Ø1

**Fig. 1:**
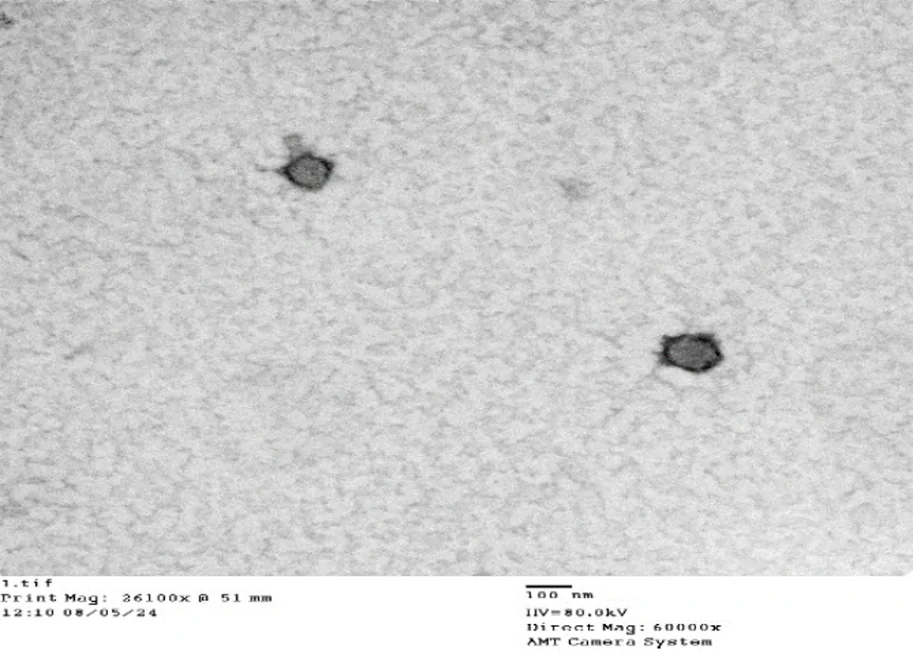
TEM of *K. pneumoniae* phage KP Ø1 using Beckman 1010 with operating voltage (80 kV), staining protocol (2% uranyl acetate, negative stain) in imaging center (Regional Center of Mycology and Biotechnology, Al-Azhar University, Cairo, Egypt).

*K. pneumoniae* phage KP Ø1 was isolated at a titer of 10^8^ and an ability to infect 50% of isolates. Its phenotypic characterization by spot test showed tiny plaques surrounded by semitransparent halos, and by Transmission Electron Microscopy (TEM) showed an icosahedral head of 92.3 nm and a thick, very short, contractile tail that was 85.1 nm in length (Fig. 1).

### Temperature and pH stability of *K. pneumoniae* phage KP Ø1

*K. pneumoniae* phage KP Ø1 was highly stable (10⁸ PFU/mL) over a wide range of temperature (-20 –50 °C); supporting its suitability for long-term biobanking and phage stock preparation, and suggesting potential activity at physiological temperature and over a pH range (7–9), although these findings reflect in vitro stability testing rather than confirmed in vivo or therapeutic performance. However, exposure to elevated temperatures induced a progressive decline in viability; a one log reduction in viral titer was observed at 60 °C, and the phage dropped below the limit of detection at 70 °C and 80 °C. Similarly, deviations from the optimal pH range led to significant inactivation. The phage exhibited a one log reduction at pH 5 and 10, and a two log reduction at pH 4. Extreme environmental conditions at the outer limits of the scale, specifically pH 1, 2, 12, and 13, completely inactivated the phage, reducing titers below detectable limits.

### Genomics of the phage *K. pneumoniae* phage KP Ø1

#### Genomic analysis of *K. pneumoniae* phage KP Ø1

Whole genome sequencing of *K. pneumoniae* phage KP Ø1 generated high-quality paired-end reads assembled into a single contig with N50 (assembly contiguity metric) value of 174,591 bp and an overall GC content of 41.86% (Accession number: PZ022450). The predicted open reading frames (ORF) in the complete genome of *K. pneumoniae* phage KP Ø1 phage were 274 genes of which were 117 functional, 98 hypothetical genes, and 59 unknown were represented in Supplementary Table S3. Genome completeness assessment using CheckV v0.8.1 classified KP Ø1 as high quality with 98.0% completeness (AAI-based, high-confidence). The assembled genome showed a contamination score of 0.0 and no assembly warnings that confirmed the integrity and purity of the sequenced genome.

#### Functional genomic annotation of *K. pneumoniae* phage KP Ø1

**Fig. 2:**
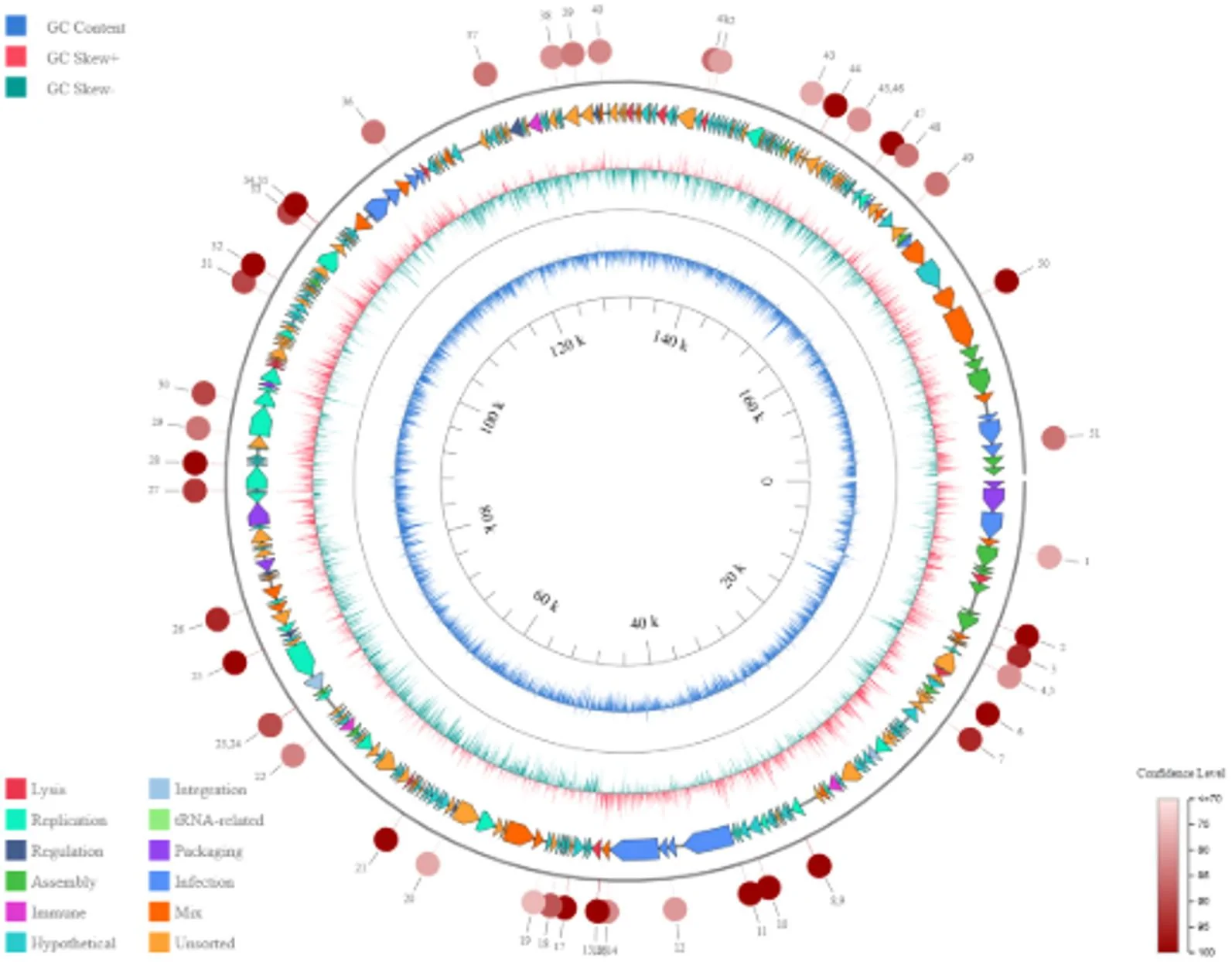
Circular genome visual map and functional annotation of *K. pneumoniae* phage KP Ø1 bacteriophage using PhageScope Platform database; Task ID: 41733; each color represent a different function of encoding protein plotted on the left bottom side with confidence level on the right side.

Genomic annotation of *K. pneumoniae* phage KP Ø1 revealed 274 predicted open reading frames (ORFs) distributed across 11 functional categories, including packaging, assembly, infection, lysis, replication, regulation, recombination, immune evasion, hypothetical proteins, proteins of unknown function, and tRNA as shown in Fig. 2, Supplementary Table S4, and Supplementary Figure S1.

Among functionally annotated ORFs, the assembly module was the most represented (*n* = 30; 10.95%), encoding structural components essential for virion morphogenesis including major capsid proteins (Gp23; Klebsiella_10, Klebsiella_11, Klebsiella_98), head scaffolding and decoration proteins (Klebsiella_7, Klebsiella_9, Klebsiella_21), tail tube proteins (Klebsiella_4, Klebsiella_173, Klebsiella_257), and baseplate subunits (Klebsiella_169, Klebsiella_266–Klebsiella_269).

Replication associated genes constituted the second largest functionally annotated category (*n* = 27; 9.85%), encompassing a predicted DNA replication apparatus including helicases (Klebsiella_16, Klebsiella_104), primase (Klebsiella_95), DNA sliding clamp components (Klebsiella_111, Klebsiella_113), ribonucleotide reductases (Klebsiella_134, Klebsiella_135), RNA and DNA ligases (Klebsiella_32, Klebsiella_138, Klebsiella_162, Klebsiella_254), and dihydrofolate reductase (Klebsiella_132). Based on this gene annotation, these ORFs are proposed to support the capacity of *K. pneumoniae* phage KP Ø1 to redirect host biosynthetic resources toward phage genome duplication, a pattern consistent with, though not functionally confirmed for, the largely host-independent replication strategy that is a hallmark of obligately lytic phages; this interpretation is based on gene annotation and has not been experimentally verified.

Infection related ORFs (*n* = 24; 8.76%) encoded the full complement of host adsorption machinery, including tail fiber proteins (Klebsiella_55–Klebsiella_58), tail fibre adhesin Gp38 (Klebsiella_59), baseplate hub and wedge proteins (Klebsiella_170–Klebsiella_175, Klebsiella_261), and a tail fiber chaperone (Klebsiella_255), mediating specific recognition of *Klebsiella pneumoniae* surface receptors, most notably the capsular polysaccharide (CPS). The specificity of these tail fiber proteins toward *K. pneumoniae* CPS receptors suggests that *K. pneumoniae* phage KP Ø1 possesses a targeted host range. Such receptor specificity is expected, in principle, to be advantageous for phage therapy applications in urinary tract infection management by limiting off-target effects on the commensal microbiota while targeting the pathogen; however, this potential benefit is inferred from genomic and receptor-binding predictions and was not experimentally assessed in the present study.

The lysis module comprised 13 ORFs (4.74%), including a holin (Klebsiella_60), multiple lytic transglycosylases (Klebsiella_88, Klebsiella_214), Rz-like spanins (Klebsiella_139, Klebsiella_140), peptidases (Klebsiella_8, Klebsiella_18, Klebsiella_208), a gene 25-like lysozyme (Klebsiella_176), a D-alanyl-D-alanine carboxypeptidase (Klebsiella_219), and lysis inhibitors RIIA and RIIB (Klebsiella_71, Klebsiella_72), indicating a tightly regulated lysis inhibition system analogous to that described in phage T4, where lysis timing is modulated in response to superinfection to maximize phage progeny yield.

Regulatory genes (*n* = 9; 3.28%) included transcriptional and translational regulators governing temporal gene expression throughout the lytic cycle, while packaging genes (*n* = 6; 2.19%) encoded the terminase complex (Klebsiella_1, Klebsiella_2) and associated endonucleases (Klebsiella_137) responsible for DNA encapsidation via a headful packaging mechanism.

Recombination-associated ORFs (*n* = 4; 1.46%), including RecA (Klebsiella_106), DprA-like recombination mediator (Klebsiella_33), UvsY recombination protein (Klebsiella_12), and Recombination Endonuclease VII (Klebsiella_41), were identified by homology-based annotation. Based on the known functions of these gene families in other phages, their presence is consistent with a possible role in recombination-dependent replication and genomic plasticity rather than in lysogeny; this functional role has not been experimentally tested in *K. pneumoniae* phage KP Ø1.

The immune evasion category (*n* = 3; 1.09%) comprised two putative DNA methyltransferases (Klebsiella_39, Klebsiella_99) and a predicted nucleotidyltransferase (Klebsiella_194). Based on homology to characterized methyltransferases in other phages, these genes are proposed to protect phage DNA from host restriction-modification (R-M) systems, which would represent an adaptation for successful infection of clinically relevant *K. pneumoniae* strains that harbor diverse R-M defenses; this proposed function is based on bioinformatic annotation and has not been experimentally confirmed for *K. pneumoniae* phage KP Ø1.

A single tRNA gene (Klebsiella_210; 0.36%) encoding a valyl-tRNA synthetase modifier was also identified, which may enhance translational efficiency by supplementing host tRNA pools that are depleted or suboptimal for phage codon usage during late stage of lytic replication.

However, the largest proportion of ORFs comprised hypothetical proteins (*n* = 98; 35.77%), a feature commonly reported across novel bacteriophage genomes and reflecting the vast reservoir of phage-specific genes that remain functionally uncharacterized, as well as the rapid evolutionary divergence of phage proteomes relative to their bacterial counterparts. An additional 59 ORFs (21.53%) were classified as proteins of unknown function, bringing the combined total of uncharacterized genetic content to over 57% of the genome. A proportion consistent with previously reported *Klebsiella* phage genomes and highlighting the considerable unexplored genetic diversity within phage populations. The high proportion of uncharacterized ORFs in *K. pneumoniae* phage KP Ø1 is not unexpected, as large T4-like phage genomes characteristically encode a substantial fraction of genus- or species- specific genes with no detectable homologs in current databases, and their functional role will require dedicated proteomic and mutagenesis studies in future work.

#### Phylogenomic analysis and taxonomic classification of *K. pneumoniae* phage KP Ø1

**Fig. 3:**
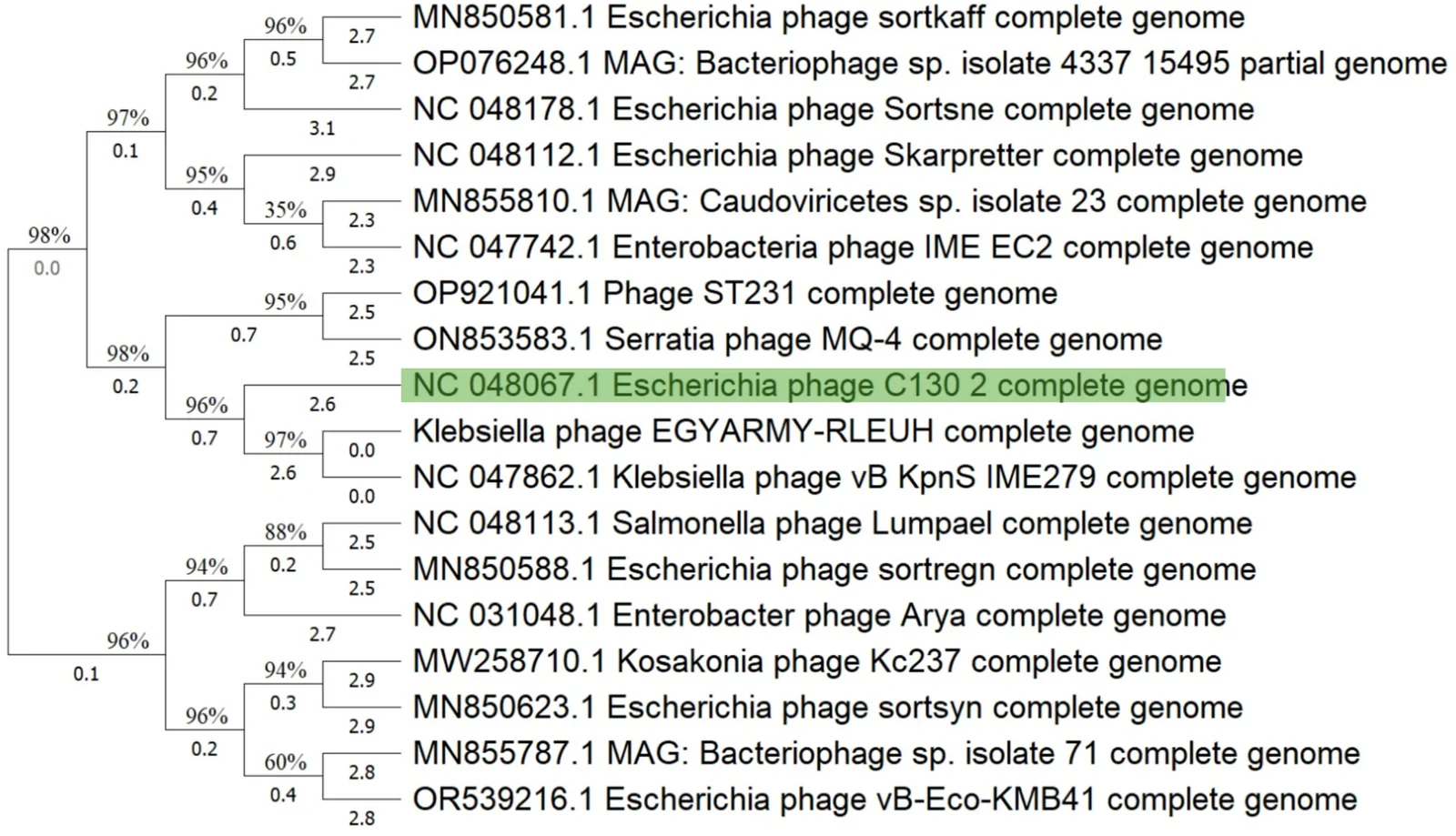
Rectangular phylogenomic tree of top matching phages with *K. pneumoniae* phage KP Ø1 (Klebsiella phage EGYARMY-RLEUH); accession number: PZ022450, highlighted in green using MEGA 12, 1000 bootstraps.

To establish the evolutionary position of *K. pneumoniae* phage KP Ø1 within the bacteriophage taxonomic framework, a whole-genome-based phylogenomic tree was constructed using sequences of related phages retrieved from the NCBI GenBank database (Fig. 3). The resulting tree demonstrated that *K. pneumoniae* phage KP Ø1 clustered within a well-supported monophyletic clade (bootstrap value = 98%), including phages infecting diverse Enterobacteriaceae hosts, including *Escherichia*, *Klebsiella*, *Serratia*, *Salmonella*, *Enterobacter*, and *Kosakonia* species; a host breadth characteristic of the family *Straboviridae*. Within this overarching clade, *K. pneumoniae* phage KP Ø1 occupied a distinct phylogenetic position with the highest evolutionary affinity to *Klebsiella *phage vB_KpnS_IME279 (bootstrap = 97%; branch length = 0.0), indicating an exceptionally high degree of genomic conservation between these two phages and supporting their placement within the same genus. The near-zero branch length uniting KP Ø1 and vB_KpnS_IME279 reflects minimal nucleotide divergence at conserved gene loci, suggesting recent common ancestry and strong purifying selection acting on core structural and replication genes. Phylogenetic analyses of closely related phages have consistently classified members of this cluster within the genus *Slopekvirus* of the family *Straboviridae*, demonstrating high sequence identity with phages infecting *Enterobacter*, *Escherichia*, and *Klebsiella* species; a pattern fully consistent with the clustering observed for *K. pneumoniae* phage KP Ø1 in the present analysis.

Taxonomic classification using the PhaBOX PhaGCN module assigned *K. pneumoniae* phage KP Ø1 to the genus *Slopekvirus* with a genus-level confidence score of 0.82, classified within a known genus cluster. The complete ICTV-compliant taxonomic lineage was determined as: Kingdom *Heunggongvirae*, Phylum *Uroviricota*, Class *Caudoviricetes*, Family *Straboviridae*, Genus *Slopekvirus*. This classification was independently corroborated by PhageScope HMM-based taxonomic annotation, which assigned *K. pneumoniae* phage KP Ø1 to class *Caudoviricetes* with a 100% hit rate across 262 HMM profile hits.

#### Host assignment

Computational host assignment using PhageScope BLAST-based analysis identified *Klebsiella pneumoniae* as the host organism, with complete taxonomic lineage: Phylum *Pseudomonadota*, Class *Gammaproteobacteria*, Order *Enterobacterales*, Family *Enterobacteriaceae*, *Genus Klebsiella*, Species *Klebsiella pneumoniae*. This result is fully consistent with experimental isolation data. PhaBOX CHERRY module predicted Escherichia at the genus level using an amino acid identity (AAI)-based method with a moderate confidence score of 0.82. This discrepancy is attributed to the conserved structural protein sequences shared among Enterobacteriaceae phages within the CHERRY training database. BLAST-based host assignment, which relies on direct sequence homology, provides more reliable host identification and corroborates experimental evidence.

#### Obligately lytic nature of *K. pneumoniae* phage KP Ø1 and therapeutic implications

Genomic analysis of *K. pneumoniae* phage KP Ø1 confirmed the complete absence of lysogeny-associated genes, including integrase, excisionase, and CI repressor-encoding sequences, establishing *K. pneumoniae* phage KP Ø1 as an obligately lytic bacteriophage incapable of establishing a lysogenic relationship with its host. This finding is fully consistent with the strictly lytic lifestyle characteristic of members of the genus *Slopekvirus* within the family *Straboviridae* and represents a critical prerequisite for safe therapeutic application. Instead, *K. pneumoniae* phage KP Ø1 encodes a complete and well-characterized lytic cassette comprising a holin (Klebsiella_60), multiple lytic transglycosylases (Klebsiella_88, Klebsiella_214), Rz-like spanins (Klebsiella_139, Klebsiella_140), and lysis inhibition regulators RIIA and RIIB (Klebsiella_71, Klebsiella_72), the latter of which also serve as canonical genomic landmarks for genome start site annotation within the *Straboviridae* in accordance with NCBI annotation conventions. However, several ORFs under the recombination category such as RecA-like recombinase and DprA-like proteins; these are functionally part of the DNA replication and repair machinery typical of *Slopekvirus* phages, rather than lysogeny determinants. *K. pneumoniae* phage KP Ø1 encodes a predicted replication apparatus that, based on gene annotation, is consistent with the largely host-independent replication strategy characteristic of obligately lytic phages, and is proposed as a further supportive indicator of its potential as a phage therapy candidate against uropathogenic multidrug-resistant *Klebsiella pneumoniae*.

Computational lifestyle prediction using PhaBOX (PhaTYP module) and PhageScope consistently classified *K. pneumoniae* phage KP Ø1 as a strictly virulent (lytic) phage with maximum confidence (score 1.0). Genomic safety assessment using PhageScope revealed that *K. pneumoniae* phage KP Ø1 encodes no virulence factors (screened against VFDB), no antimicrobial resistance genes (screened against CARD database), and no anti-CRISPR proteins. Additionally, no CRISPR arrays were detected within the KP Ø1 genome itself. Combined with the absence of lysogeny-related genes confirmed by dual-tool lifestyle prediction, these findings establish the genomic safety profile of *K. pneumoniae* phage KP Ø1 as a candidate for therapeutic applications.

### Statistical analysis

All stability assays were performed using duplicate technical spots (*n* = 2 spots per condition) in a single experimental session, consistent with the double-layer standard spot assay methodology described in Sect. 3.3. Phage titers at each condition are reported as the mean of the two duplicate spot values (PFU/mL), expressed on a log₁₀ scale. The range between duplicate spot values is reported as a measure of technical reproducibility. Host range percentages were calculated as the number of susceptible isolates divided by the total number of isolates tested, expressed as a percentage, with no inferential statistical testing applied to proportional data given the descriptive nature of the host range assessment.

## Discussion

Urinary tract infections (UTIs) are mainly implicated by GNB that are becoming an evolving threat to public health because of their ability to acquire genes, located on transferable plasmids and that code for multiple enzymes that confer drug resistance^21^. Although the symptoms of UTIs are heterogeneous and range from uncomplicated (uUTIs) to complicated (cUTIs), most UTIs are usually treated empirically. Resistant strains develop as a result of misuse of antibiotics where bacteria can acquire resistance genes horizontally. Acquisition of resistance genes under antibiotic selection pressure and dissemination of these genes lead to the development of multidrug resistant (MDR) and extensive drug resistant (XDR) strains which confronts antimicrobial chemotherapy universally^22^.

Development of new antibiotic classes has slowed with very limited newly introduced therapeutic options. This has led to reviving of an older approach to treat infections via phages^16^. Bacteriophages, unlike antibiotics have special features such as the relative abundance; playing crucial roles in regulating bacterial populations and influencing microbial ecosystems, host specificity; thus, do not impact other commensals, shorter time of treatment and antibiofilm activity. Moreover, they have the unique ability to replicate at the sites of infection and usually have low toxicity^21^.

Our study was conducted on 43 out of 50 (86%) *K. pneumoniae* and 7 out of 50 (14%) *E. coli* MDR uropathogens. *K. pneumoniae* isolates were resistant (100%) to piperacillin/tazobactam (TZP), ampicillin/sulbactam (SAM), ceftazidime (CAZ), ceftriaxone (CRO), cefoxitin (FOX), cefepime (FEP), cefazolin (CFZ), meropenem (MEM), ciprofloxacin (CIP), nitrofurantoin (F) and trimethoprim/sulfamethoxazole (SXT), while *E.coli* isolates were resistant (100%) to ampicillin (AMP), ampicillin/sulbactam (SAM), ceftazidime (CAZ), ceftriaxone (CRO), cefoxitin (FOX), cefepime (FEP), cefazolin (CFZ), tobramycin (TOB), ciprofloxacin (CIP), levofloxacin (LEV) and trimethoprim/sulfamethoxazole (SXT). *K.pneumoniae* isolates were resistant to amikacin (AK), gentamicin (CN), tobramycin (TOB) and levofloxacin (LEV) (88.4%, 60%, 98%, 98% respectively), while *E. coli* isolates were resistant to TZP, MEM, AK, CN and F (86%, 86%, 29%, 71%, 29% respectively). This was concordant as regard antibiotic resistance with *Jalil and Al Atbee in 2022* who showed that *K. pneumoniae* isolates were resistant to LEV, SXT, CAZ and CN (78.9%, 71.1%, 65.8% and 63.2% respectively), and *E. coli* isolates were resistant to CRO, AMP, LEV, CAZ, CN and SXT (89.0%, 86.6%, 82.9%, 68.3% 54.9%, and 53.7% respectively). Also, Gebretensaie et al.^23^ observed that *K. pneumoniae* showed high resistance to CIP, STX (100%) and CFZ (80%) while *E. coli* showed high resistance to AMP, SAM, STX and CFZ (98%, 100%, 80% and 65.3%). However, it was discordant as regard predominant uropathogen where Jalil and Al Atbee^24^ stated that the most prevalent uropathogen was *E. coli* which was found in 82 out of 120 (68.3%) cases followed by *K. pneumoniae* found in 38 out of 120 (31.7%). Also, Islam et al.^25^ stated that *E. coli* (41.66%), *Enterococcus faecalis* (23.48%) and *K. pneumoniae* (18.93%) were the predominant uropathogens. Predominance of *K. pneumoniae* as a uropathogen in our study may be attributed to its ability to cause hospital-acquired UTIs predominantly in elderly, newborn, critically ill and immunocompromised patients.

In our study, ten bacteriophages specific to *E. coli* and three phages specific to *K. pneumoniae* were isolated from sewage water collected from different places and produced lysis with their bacterial host. Only *K. pneumoniae* phage KP Ø1 has ability to infect 50% (25/50) of isolates So it has the broadest host range in comparison with other phages *E.coli* phage EC Ø1, *E.coli* phage EC Ø2, *E.coli* phage EC Ø3, *E.coli* phage EC Ø4, *E.coli* phage EC Ø5, *E.coli* phage EC Ø6, *E.coli* phage EC Ø7, *E.coli* phage EC Ø8, *E.coli* phage EC Ø9, *E.coli* phage EC Ø10, *K. pneumoniae* phage KP Ø2 and *K. pneumoniae* phage KP Ø3 that were able to infect (7 (14%), 3 (6%), 3 (6%), 7 (14%), 5 (10%), 4 (8%), 5 (10%), 3 (6%), 4 (8%), 4 (8%), 8 (16%) and 17 (34%) respectively. This was similar to Concha-Eloko et al.^26^ who stated that two new *Klebsiella* phages isolated from the environment, vB_Kpn_K7PH164C4 and vB_Kpn_K30λ2.2 had broad host range which infected strains of more than 20 different capsular types representing the broadest infection range observed for *Klebsiella* phages. This was in disagreement with Fang et al.^27^ who stated that three novel lytic phages vB_KpnA_SCNJ1-Z, vB_KpnS_SCNJ1-C and vB_KpnM_SCNJ1-Y against a CR-hvKP strain SCNJ1 showed a narrow host range only lysing 1 of 50 bacterial strains.

Our study showed that *K. pneumoniae* phage KP Ø1 forms tiny plaques surrounded by halos and belongs to *Slopekvirus* genus possessing an icosahedral heads, and thick short and contractile tails. This was concordant with Townsend et al.^28^ who stated that a novel phage vB_KpM-Mild isolated from sewage for lysis of *K. pneumoniae* belonged to *Slopekvirus* genus. However, discordant with Zhang et al.^29^ who stated that a novel phage vB_KpnP_IME279 isolated from hospital sewage for lysis of MDR *K.pneumoniae* isolated from urine belonged to *Podoviridae* family.

Phage stability under varying environmental conditions is a critical determinant of practical therapeutic utility, directly influencing storage requirements, transport logistics, formulation design, and in vivo activity at the site of infection. Our study revealed that *K. pneumoniae* phage KP Ø1 was completely thermal stable across a wide temperature range of − 20 °C to 50 °C after 1 h incubation, with no detectable reduction in phage titer across this range (10⁸ PFU/mL) to be fully consistent with its biological classification as a large dsDNA myobacteriophage of the genus *Slopekvirus*, family *Straboviridae*, whose structural proteins are architecturally optimized for stability under the physiological conditions encountered during *K. pneumoniae* infection. Stability at − 20 °C was specifically assessed to confirm suitability for long-term biobanking and phage stock preparation, as this represents the standard temperature for long-term storage of therapeutic phage preparations; the confirmed stability at this temperature establishes that *K. pneumoniae* phage KP Ø1 stocks can be maintained without titer loss during prolonged storage prior to clinical use. Stability at 4 °C confirms suitability for standard refrigerated transport and short-term clinical storage, while stability at 37 °C (human physiological body temperature) confirms that *K. pneumoniae* phage KP Ø1 retains full lytic activity at this physiologically relevant temperature in vitro, supporting, though not confirming, its potential to remain active under in vivo conditions; direct evaluation in a biological system will be required to establish this as a prerequisite for therapeutic application. The thermostability of *K. pneumoniae* phage KP Ø1 across this range reflects the structural integrity of its large icosahedral capsid formed by the cross-linked major capsid protein Gp23 (Klebsiella_10, Klebsiella_11, Klebsiella_98), which undergoes extensive post-translational conformational stabilization. Exposure to elevated temperatures induced a progressive and biologically predictable decline in viability: a one-log reduction in phage titer was observed at 60 °C, attributable to the onset of thermal denaturation of tail fiber proteins (Klebsiella_55–Klebsiella_58) and the Gp38 tail fibre adhesin (Klebsiella_59), which are the most thermolabile structural components. The phage titer was below the limit of detection (ND) at 70 °C and 80 °C, consistent with irreversible denaturation of capsid proteins, dissociation of tail fibers from the baseplate, and structural collapse of the head-tail junction proteins including the head-tail adaptor (Klebsiella_181) at these temperatures.

The thermal stability profile of *K. pneumoniae* phage KP Ø1 (stable at − 20 °C to 50 °C) is broadly concordant with previously characterized *Klebsiella*-infecting myophages. Chen,^30^ stated that phage vB_KpP_HS106 isolated from hospital sewage against MDR hypervirulent *K. pneumoniae*, remained stable across 4 °C to 50 °C. Similarly, Parra et al.^31^ reported that *Slopekvirus* phage KpCCP1 demonstrated stability at 4 °C to 50 °C; a finding of particular relevance as KpCCP1 belongs to the same genus as *K. pneumoniae* phage KP Ø1, confirming that the observed thermal stability range is a genus-level characteristic of *Slopekvirus* members rather than an idiosyncratic property of *K. pneumoniae* phage KP Ø1. Discordantly, Fang et al.^27^ reported that phages vB_KpnA_SCNJ1-Z, vB_KpnS_SCNJ1-C, and vB_KpnM_SCNJ1-Y maintained stability up to 60 °C, and Pu et al.^32^ reported that phage BUCT541 retained activity at 60 °C and 70 °C. These discrepancies are attributable to genus- and family-level differences in major capsid protein structure and cross-linking density rather than contradicting the findings for *K. pneumoniae* phage KP Ø1; phages belonging to different *Straboviridae* genera with structurally distinct Gp23 variants naturally exhibit different upper thermal stability thresholds, and the narrower thermal range of *K. pneumoniae* phage KP Ø1 relative to these comparators is fully consistent with its *Slopekvirus* classification.

Regarding pH stability, *K. pneumoniae* phage KP Ø1 demonstrated complete stability within the range of pH 7–9, with no reduction in phage titer observed across these conditions (10⁸ PFU/mL). The optimal pH stability range of pH 7–9 precisely mirrors the intracellular physiological pH of *K. pneumoniae* (~ 7.2–7.6) and the pH of human blood (7.35–7.45)^2^. This overlap suggests that *K. pneumoniae* phage KP Ø1 possesses structural proteins that remain stable and active under the exact physicochemical conditions encountered during bacterial infection, consistent with, though not direct evidence of, adaptation to these conditions; extrapolation to performance during systemic therapeutic application would require further experimental support. Progressive pH-dependent inactivation was observed as conditions deviated from this optimal range: a one-log titer reduction was recorded at pH 5 and pH 10, reflecting partial protonation and deprotonation respectively of ionizable amino acid residues within tail fiber and capsid surface proteins, reducing structural integrity and adsorption efficiency without complete virion destruction. A two-log titer reduction at pH 4 reflects more pronounced partial denaturation of the Gp38 tail fibre adhesin (Klebsiella_59) and tail fiber tip proteins responsible for CPS receptor binding, as these surface-exposed proteins contain ionizable residues that are particularly sensitive to acidic conditions. Complete inactivation below the limit of detection was observed at extreme pH values (pH 1, 2, 12, and 13), consistent with irreversible acid-mediated protonation and hydrolysis of capsid protein peptide bonds at extreme acidity, and hydroxide-mediated hydrolysis and charge repulsion-driven dissociation of capsid subunits at extreme alkalinity; processes that result in permanent loss of virion structural integrity and release of phage DNA from the capsid head.

The pH stability profile of *K. pneumoniae* phage KP Ø1 is concordant with phage vB_KleM_KB2 reported by Peng et al.^33^, which demonstrated high lytic activity against *K. pneumoniae* within pH 6–9, overlapping with the optimal stability range of KP Ø1. By contrast, Fang et al.^27^ and Pu et al.^32^ reported broader pH tolerance (pH 3–14 and pH 3–11 respectively) for phages of different *Klebsiella*-infecting genera; differences attributable to genus-specific differences in capsid protein surface charge distribution and structural robustness rather than representing a limitation of *K. pneumoniae* phage KP Ø1.

Critically, the pH stability range of *K. pneumoniae* phage KP Ø1 (pH 7–9) encompasses the full clinically relevant pH range for therapeutic application in UTI. In UTI patients infected with urease-producing *K. pneumoniae* strains, urinary pH is typically shifted toward alkalinity (pH 6.5–8.0) due to urease-mediated conversion of urea to ammonia, conditions under which *K. pneumoniae* phage KP Ø1 retains complete lytic activity. While *K. pneumoniae* phage KP Ø1 demonstrates a one-log reduction at pH 5, this moderately acidic condition is encountered only in highly concentrated or post-hydration urine rather than at the site of active *K. pneumoniae* infection, and the residual titer of 10⁷ PFU/mL at pH 5 remains above the therapeutic threshold typically considered sufficient for phage therapy efficacy. Collectively, the thermal and pH stability profiles of *K. pneumoniae* phage KP Ø1 support its suitability for standard clinical storage (− 20 °C and 4 °C) and suggest activity under conditions approximating physiological in vivo parameters (37 °C, pH 7–9) and compatibility with the urinary microenvironment; these remain in vitro observations, and actual therapeutic application against uropathogenic MDR *K. pneumoniae* remains to be established through future in vivo studies.

Our study revealed the genomic and phylogenomic characterization of *K. pneumoniae* phage KP Ø1, a novel obligately lytic bacteriophage isolated against uropathogenic multidrug-resistant *Klebsiella pneumoniae*, and provides comprehensive evidence supporting its formal classification within the genus *Slopekvirus*, family *Straboviridae*. Whole-genome sequencing and annotation of *K. pneumoniae* phage KP Ø1 revealed a large dsDNA genome of size 174,591 bp encoding 274 predicted ORFs distributed across 11 functional categories, encompassing the genes predicted to encode a complete lytic machinery including DNA packaging, virion assembly, host infection, genome replication, lysis, and regulatory modules; a genomic architecture fully consistent with other characterized *Slopekvirus* members. Moreover, the identification of putative DNA methyltransferases and a predicted nucleotidyltransferase within the immune evasion module raises the possibility, based on genome annotation alone, that *K. pneumoniae* phage KP Ø1 may possess mechanisms to circumvent host restriction-modification defense systems, which could theoretically enhance its capacity to infect clinical *K. pneumoniae* isolates harboring diverse defense arsenals; this proposed function is a bioinformatic prediction that has not been experimentally validated. *Slopekvirus* phages are characterized by their large genomes encoding specialized machinery to overcome bacterial defenses, a trait reflected in the diverse functional repertoire of KP Ø1. This was concordant with Parra et al.^31^ who stated that The phage KpCCP1 consists of a 177,276 bp double-stranded DNA molecule, with a GC content of 41.82%, further supporting the correct taxonomic placement of KP Ø1 within this genus, but discordant with Mulani et al.^34^ who showed that genome sequence of phage PG14 consists of 49,853 bp with a GC content of 50.8% and this was related to the family and the genus that phage belongs to.

Phylogenomic analysis demonstrated that *K. pneumoniae* phage KP Ø1 clustered within a well-supported monophyletic clade (bootstrap = 98%) alongside phages infecting diverse Enterobacteriaceae hosts, with the highest evolutionary affinity to *Klebsiella *phage vB_KpnS_IME279 (bootstrap = 97%; branch length = 0.0), consistent with classification within the genus *Slopekvirus.*

Although the PhaBOX CHERRY module predicted Escherichia as a potential host (score 0.82), this is attributed to the high conservation of structural proteins across *Enterobacteriaceae* phages, which often leads to AAI-based misclassification. In contrast, BLAST-based analysis and direct sequence homology results from PhageScope correctly identified *Klebsiella pneumoniae*, which is fully consistent with our experimental isolation data.

The strictly lytic lifestyle of *K. pneumoniae* phage KP Ø1 was confirmed by two independent computational tools with maximum confidence scores (PhaBOX PhaTYP score: 1.0; PhageScope lifestyle prediction: 100% virulent). CheckV analysis confirmed the absence of proviral elements with zero contamination score, collectively establishing the strictly lytic nature of *K. pneumoniae* phage KP Ø1. The annotation of several ORFs under the ‘recombination’ functional category such as: genes encoding RecA-like recombinases, DprA-like mediator proteins, and recombination endonucleases are well-documented components of the phage DNA replication and repair machinery, functioning to facilitate recombination-dependent replication and genome maintenance rather than chromosomal integration. The complete absence of lysogeny-associated genes, including integrase, excisionase, attP attachment sites and CI repressor-encoding sequences, confirms the obligately lytic nature of *K. pneumoniae* phage KP Ø1, fulfilling a fundamental biosafety criterion for potential of therapeutic application.

The absence of virulence factors, antimicrobial resistance genes, anti-CRISPR proteins, and lysogeny determinants, confirmed by multiple databases (VFDB, CARD) and computational tools, meets the minimum genomic requirements for therapeutic phage candidates as outlined in current phage therapy guidelines. However, it must be acknowledged that genomic safety screening, while necessary, is not sufficient to establish therapeutic safety and efficacy. Functional characterization including one-step growth kinetics, adsorption assays, burst size determination, biofilm degradation potential, and in vivo efficacy studies in appropriate animal models are essential next steps before clinical application can be considered. The present study establishes the genomic foundation for such future investigations.

## Conclusion

The genomic, and phylogenomic characteristics of *K. pneumoniae* phage KP Ø1, including its obligately lytic lifestyle, a genome architecture predicted to support largely host-independent replication, host-range specificity that may reduce off-target effects on the commensal microbiota (a genomically inferred property warranting experimental confirmation), absence of virulence- or antibiotic resistance-associated genes, and evolutionary novelty at the species level, denote it as a promising candidate for future functional characterization and in vivo animal model studies, and investigating of *K. pneumoniae* phage KP Ø1 as a component of phage cocktails to broaden therapeutic coverage and overcome the emergence of phage resistance before any clinical application for the treatment of *K. pneumoniae* related infections. The genomic data generated in this study further contribute to the expanding repository of characterized *Klebsiella*-infecting *Slopekvirus* phages, supporting future comparative genomic and evolutionary studies of this therapeutically relevant phage genus. It is crucial to continue research on molecular testing for genes of bacterial resistance to show its effect on phage-host interaction, also, future studies should assess whether *K. pneumoniae* phage KP Ø1 demonstrates antibiofilm activity and whether it can augment treatment of MDR pathogens in clinical settings.

### Limitations

Several limitations of the present study should be acknowledged. First, functional characterization experiments including one-step growth curves, adsorption kinetics, burst size determination, efficiency of plating, and biofilm degradation assays were not performed in this study, represent important areas for future investigation, and are planned for subsequent studies. Second, host range determination was conducted against 50 clinical isolates, and evaluation against larger, more diverse collections would provide a more comprehensive assessment of *K. pneumoniae* phage KP Ø1 breadth of infection. Third, no animal studies or ex vivo infection models were employed, the therapeutic potential described here remains preliminary and genomically inferred rather than experimentally demonstrated and in need for further clinical trials for in vivo assessment. Fourth, comparative genomic analysis including average nucleotide identity (ANI) values, whole-proteome comparisons with closely related *Slopekvirus* members could not be completed within the scope of this work and are planned for subsequent studies.

## Supplementary Information

Below is the link to the electronic supplementary material.

Supplementary Material 1

## Data Availability

The authors declare that data generated and/or analyzed during the current study are available in the NCBI repository (https://share.google/WxI1kJrIwS8WHPJ2i) with accession number: PZ022450.

## Abbreviations

MDR: Multi Drug Resistant
GNB: Gram negative bacteria
GN: Gram negative
*K. pneumoniae*: *Klebsiella pneumoniae*
KP Ø1: *K. pneumoniae* phage − 01
TEM: Transmission Electron Microscopy
dsDNA: Double-stranded deoxyribonucleic acid
WGS: Whole-genome sequencing
ORFs: Open reading frames
CDS: Coding Sequence
AMR: Antimicrobial resistance genes
UTIs: Urinary tract infections
*E. coli*: *Escherichia coli*
*P. mirabilis*: *Proteus mirabilis*
AST: Antimicrobial susceptibility testing
CLSI: Clinical & Laboratory Standards Institute
TSB: Tryptic soy broth
2xNB: Double strength nutrient broth
PVDF: Polyvinylidene fluoride
SM buffer: Sodium Magnesium buffer
DAO: Double agar overlay
RT: Room temperature
PFU/ml: Plaque forming unit per ml
h: Hour
Hcl: Hydrochloric acid
NaOH: Sodium hydroxide
DW: Distilled water
SDS: Sodium Dodecyl Sulfate
EDTA: Ethylenediaminetetraacetic acid
RAST: Rapid Annotation using Subsystem Technology
BLASTp: Basic Local Alignment Search Tool
NCBI: National Center of Biotechnology Information
MUSCLE: Multiple Sequence Comparison by Log-Expectation
ICTV: International Committee on Taxonomy of Viruses
MEGA: Molecular Evolutionary Genetics Analysis
N50: Assembly contiguity metric
GC content: Guanine Cytosine content
ESBLs: Extended spectrum β-lactamases
VFDB: Virulence Factor Database
CARD: Comprehensive Antibiotic Resistance Database
CRISPR: Clustered Regularly Interspaced Short Palindromic Repeats
uUTIs: Uncomplicated Urinary tract infections
cUTIs: Complicated Urinary tract infections
TZP: Piperacillin/tazobactam
SAM: Ampicillin/sulbactam
CAZ: Ceftazidime
CRO: Ceftriaxone
FOX: Cefoxitin
FEP: Cefepime
CFZ: Cefazolin
MEM: Meropenem
CIP: Ciprofloxacin
F: Nitrofurantoin
SXT: Trimethoprim/sulfamethoxazole
AMP: Ampicillin
TOB: Tobramycin
LEV: Levofloxacin
AK: Amikacin
CN: Gentamicin
EC Ø1: E.coli phage-01
Bp: Base pair
tmRNA: Transfer-messenger ribonucleic acid
tRNA: Transfer ribonucleic acid
AAI: Amino acid identity
XDR: Extensive drug resistant
ND: Not detected
AI: Artificial intelligence
