# Supplementary material for: Genomic and phenotypic characterization of Klebsiella pneumoniae phage KP Ø1: a novel lytic Slopekvirus targeting uropathogenic multidrug-resistant Klebsiella pneumoniae
Source: Sci Rep. 2026 Sep 3;16:27778. doi: 10.1038/s41598-026-67695-4 (PMC13545231; doi:10.1038/s41598-026-67695-4)

Supplementary Table S1: Antimicrobial resistance pattern of 50 isolates over different antibiotics

Isolates	Antibiotic	Sensitive	%	Intermediate	%	Resistant	%
E.coli	AMP	-	-	-	-	7	100
	TZP	-	-	1	14	6	86
	SAM	-	-	-	-	7	100
	CAZ	-	-	-	-	7	100
	CRO	-	-	-	-	7	100
	FOX	-	-	-	-	7	100
	FEP	-	-	-	-	7	100
	CFZ	-	-	-	-	7	100
	MEM	1	14	-	-	6	86
	AK	5	71	-	-	2	29
	CN	2	29	-	-	5	71
	TOB	-	-	-	-	7	100
	CIP	-		-	-	7	100
	LEV	-	-	-	-	7	100
	F	1	14	4	57	2	29
	SXT	-	-	-	-	7	100
	-	-	-	-	-	-	-
K.pneumoniae	TZP	-	-	-	-	43	100
	SAM	-	-	-	-	43	100
	CAZ	-	-	-	-	43	100
	CRO	-	-	-	-	43	100

Isolates	Antibiotic	Sensitive	%	Intermediate	%	Resistant	%
	FOX	-	-	-	-	43	100
	FEP	-	-	-	-	43	100
	CFZ	-	-	-	-	43	100
	MEM	-	-	-	-	43	100
	AK	4	9.3	1	2.3	38	88.4
	CN	6	14	11	26	26	60
	TOB	-	-	1	2	42	98
	CIP	-	-	-	-	43	100
	LEV	-	-	1	2	42	98
	F	-	-	-	-	43	100
	SXT	-	-	-	-	43	100

Abbreviations are as follow: *Klebsiella pneumoniae (K.pneumoniae)*, *Escherichia coli (E.coli),* Ampicillin (AMP), Piperacillin/Tazobactam (TZP), Ampicillin/Sulbactam (SAM), Ceftazidime (CAZ), Ceftriaxone (CRO), Cefoxitin (FOX), Cefipime (FEP), Cefazolin (CFZ), Meropenem (MEM), Amikacin(AK), Gentamicin(CN), tobramycin (TOB), Ciprofloxacin (CIP), levofloxacin (LEV), Nitrofurantoin(F), and Trimethoprim/Sulfamethaxone (SXT).

Supplementary Table S2: The isolated phages and their host range:

Phages (Ø)	Bacterial host	Host range	%
EC Ø$_1$	*EC* 1	7	14
EC Ø$_2$	*EC* 1	3	6
EC Ø$_3$	*EC* 1	3	6
EC Ø$_4$	*EC* 3	7	14
EC Ø$_5$	*EC* 1	5	10
EC Ø$_6$	*EC* 1	4	8
EC Ø$_7$	*EC* 1	5	10
EC Ø$_8$	*EC* 1	3	6
EC Ø$_9$	*EC* 1	4	8
EC Ø$_{10}$	*EC* 2	4	8
KP Ø$_1$	*KP* 24	25	50
KP Ø$_2$	*KP* 13	8	16
KP Ø$_3$	*KP* 2	17	34

Abbreviations are as follow: *Klebsiella pneumoniae (KP)*, *Escherichia coli (EC)*.

Supplementary Table S3: Functional ORF Category Summary of *Klebsiella pneumoniae phage* KP Ø1:

Functional Category	Number of ORFs	Percentage (%)
Assembly	30	10.95%
Replication	27	9.85%
Infection	24	8.76%
Lysis	13	4.74%
Regulation	9	3.28%
Packaging	6	2.19%
Recombination	4	1.46%
Immune	3	1.09%
tRNA	1	0.36%
Hypothetical	98	35.77%
Unknown	59	21.53%
total	274	100%

Supplementary Table S4: Genomic annotation of *Klebsiella pneumoniae phage* KP Ø1 revealed 274 predicted open reading frames (ORFs) encoding packaging, lysis, infection, assembly, regulation, immune and recombination.

Protein ID	Function classification	Protein Product	Function Prediction Source	Start Point	End Point	Strand
Klebsiella_1	packaging	Terminase DNA packaging enzyme	eggNOG-mapper	1	540	+
Klebsiella_2	packaging	Terminase-like family	eggNOG-mapper	509	2338	+
Klebsiella_3	infection	Phage tail sheath protein	eggNOG-mapper	2358	4349	+
Klebsiella_4	assembly infection	T4-like virus tail tube protein gp19	eggNOG-mapper	4398	4883	+
Klebsiella_5	assembly	Bacteriophage T4-like capsid assembly protein (Gp20)	eggNOG-mapper	4939	6513	+
Klebsiella_6	assembly	Pfam:Gp67	eggNOG-mapper	6513	6755	+
Klebsiella_7	assembly	head scaffolding protein	Iterative search	6765	7169	+
Klebsiella_8	lysis	Pfam:Peptidase_S77	eggNOG-mapper	7172	7819	+
Klebsiella_9	assembly	head scaffolding protein	Iterative search	7852	8640	+
Klebsiella_10	assembly	Major capsid protein Gp23	eggNOG-mapper	10000	10228	+
Klebsiella_11	assembly	Major capsid protein Gp23	eggNOG-mapper	10316	11605	+
Klebsiella_12	recombination replication	Recombination, repair and ssDNA binding protein UvsY	eggNOG-mapper	11910	12329	+
Klebsiella_13	unsorted	Protein of unknown function (DUF2685)	eggNOG-mapper	12329	12505	+
Klebsiella_14	unsorted	Unknown	-	12513	12635	+
Klebsiella_15	unsorted	Unknown	-	12583	12711	+
Klebsiella_16	replication	ATP-dependant DNA helicase UvsW	eggNOG-mapper	13110	13343	-
Klebsiella_17	unsorted	Type III restriction enzyme, res subunit	eggNOG-mapper	13352	14851	-
Klebsiella_18	lysis	peptidase activity	eggNOG-mapper	14922	15500	+
Klebsiella_19	unsorted	Unknown	-	15392	15844	-
Klebsiella_20	hypothetical	hypothetical protein	eggNOG-mapper	15932	16501	+

Klebsiella_21	assembly	Hoc-like head decoration	Iterative search	16530	17063	+
Klebsiella_22	unsorted	hydrolase activity	eggNOG-mapper	17074	17349	+
Klebsiella_23	unsorted	5'-3' deoxyribonucleotidase	Iterative search	17363	17881	+
Klebsiella_24	unsorted	5'-3' deoxyribonucleotidase	Iterative search	17914	18378	-
Klebsiella_25	hypothetical	hypothetical protein	eggNOG-mapper	18428	19267	-
Klebsiella_26	hypothetical	hypothetical protein	eggNOG-mapper	20000	20257	+
Klebsiella_27	hypothetical	hypothetical protein	eggNOG-mapper	20290	20607	+
Klebsiella_28	hypothetical	hypothetical protein	eggNOG-mapper	20604	20780	+
Klebsiella_29	hypothetical	hypothetical protein	eggNOG-mapper	20777	21103	+
Klebsiella_30	unsorted	Protein of unknown function (DUF2774)	eggNOG-mapper	21105	21461	+
Klebsiella_31	unsorted	decoy of host sigma70	Iterative search	21461	21985	+
Klebsiella_32	replication	RNA ligase	eggNOG-mapper	21989	23008	+
Klebsiella_33	recombination	DprA-like DNA recombination-mediator protein	Iterative search	22998	23579	+
Klebsiella_34	hypothetical	hypothetical protein	eggNOG-mapper	23573	24091	+
Klebsiella_35	hypothetical	hypothetical protein	eggNOG-mapper	24169	24429	+
Klebsiella_36	hypothetical	hypothetical protein	eggNOG-mapper	24439	24888	+
Klebsiella_37	unsorted	nicotinate-nucleotide diphosphorylase (carboxylating) activity	eggNOG-mapper	24916	26349	+
Klebsiella_38	hypothetical	hypothetical protein	eggNOG-mapper	26336	26638	+
Klebsiella_39	immune	D12 class N6 adenine-specific DNA methyltransferase	eggNOG-mapper	26635	27486	+
Klebsiella_40	replication	protein disulfide oxidoreductase activity	eggNOG-mapper	27495	27770	+
Klebsiella_41	recombination packaging	Recombination endonuclease VII	eggNOG-mapper	27802	28284	+
Klebsiella_42	unsorted	Unknown	-	28281	28607	+
Klebsiella_43	replication	ribonucleoside-triphosphate reductase activity	eggNOG-mapper	30000	30727	+

Klebsiella_44	hypothetical	hypothetical protein	eggNOG-mapper	30795	31037	+
Klebsiella_45	hypothetical	hypothetical protein	eggNOG-mapper	31034	31531	+
Klebsiella_46	hypothetical	hypothetical protein	eggNOG-mapper	31531	31737	+
Klebsiella_47	unsorted	Unknown	-	31758	32009	+
Klebsiella_48	hypothetical	hypothetical protein	eggNOG-mapper	32070	32264	+
Klebsiella_49	hypothetical	hypothetical protein	eggNOG-mapper	32261	32788	+
Klebsiella_50	replication	4 iron, 4 sulfur cluster binding	eggNOG-mapper	32785	33276	+
Klebsiella_51	hypothetical	hypothetical protein	eggNOG-mapper	33346	34305	+
Klebsiella_52	hypothetical	hypothetical protein	eggNOG-mapper	34348	35016	+
Klebsiella_53	replication	protein disulfide oxidoreductase activity	eggNOG-mapper	34976	35257	+
Klebsiella_54	hypothetical	hypothetical protein	eggNOG-mapper	35254	35553	+
Klebsiella_55	infection	tail fiber protein proximal subunit	Iterative search	35633	39406	+
Klebsiella_56	infection	long tail fiber protein proximal connector	Iterative search	40000	40530	+
Klebsiella_57	infection	Phage T4 tail fibre	eggNOG-mapper	40580	41248	+
Klebsiella_58	infection	virus tail, fiber	eggNOG-mapper	41257	44955	+
Klebsiella_59	assembly infection	Phage tail fibre adhesin Gp38	eggNOG-mapper	44995	45522	+
Klebsiella_60	lysis	Bacteriophage T holin	eggNOG-mapper	45639	46286	+
Klebsiella_61	hypothetical	hypothetical protein	eggNOG-mapper	46287	46556	-
Klebsiella_62	hypothetical	hypothetical protein	eggNOG-mapper	46553	46843	-
Klebsiella_63	hypothetical	hypothetical protein	eggNOG-mapper	46843	47640	-
Klebsiella_64	unsorted	Unknown	-	47721	47948	-
Klebsiella_65	hypothetical	hypothetical protein	eggNOG-mapper	47945	48274	-
Klebsiella_66	hypothetical	hypothetical protein	eggNOG-mapper	48249	48386	-
Klebsiella_67	hypothetical	hypothetical protein	eggNOG-mapper	48383	48613	-
Klebsiella_68	hypothetical	hypothetical protein	eggNOG-mapper	48658	48906	-
Klebsiella_69	unsorted	T4-like phage nuclear disruption protein (Ndd)	eggNOG-mapper	48943	49380	-

Klebsiella_70	hypothetical	hypothetical protein	eggNOG-mapper	49355	49729	-
Klebsiella_71	lysis regulation	RIIB lysis inhibitor	Iterative search	50000	50674	-
Klebsiella_72	lysis regulation	RIIA lysis inhibitor	Iterative search	50674	52935	-
Klebsiella_73	unsorted	Unknown	-	52946	53182	-
Klebsiella_74	unsorted	SprT-like family	eggNOG-mapper	53186	53740	-
Klebsiella_75	replication	DNA gyrase/topoisomerase IV, subunit A	eggNOG-mapper	53812	55098	-
Klebsiella_76	unsorted	DNA gyrase B	eggNOG-mapper	55098	56996	-
Klebsiella_77	regulation	Regulatory protein, FmdB family	eggNOG-mapper	57064	57228	-
Klebsiella_78	unsorted	unknown function	Iterative search	57228	57668	-
Klebsiella_79	hypothetical	hypothetical protein	eggNOG-mapper	57691	58275	-
Klebsiella_80	hypothetical	hypothetical protein	eggNOG-mapper	58262	58573	-
Klebsiella_81	hypothetical	hypothetical protein	eggNOG-mapper	58537	59133	-
Klebsiella_82	hypothetical	hypothetical protein	eggNOG-mapper	59173	59502	-
Klebsiella_83	hypothetical	hypothetical protein	eggNOG-mapper	59562	59786	-
Klebsiella_84	unsorted	Unknown	-	60000	60123	-
Klebsiella_85	hypothetical	hypothetical protein	eggNOG-mapper	60212	60514	-
Klebsiella_86	unsorted	Unknown	-	60511	60735	-
Klebsiella_87	unsorted	Unknown	-	60739	60888	-
Klebsiella_88	lysis	lytic transglycosylase activity	eggNOG-mapper	60952	61233	-
Klebsiella_89	unsorted	Pfam:DUF5051	eggNOG-mapper	61230	61901	-
Klebsiella_90	hypothetical	hypothetical protein	eggNOG-mapper	61898	62182	-
Klebsiella_91	hypothetical	hypothetical protein	eggNOG-mapper	62183	62491	-
Klebsiella_92	unsorted	AAA domain	eggNOG-mapper	62516	63844	-
Klebsiella_93	hypothetical	hypothetical protein	eggNOG-mapper	63841	64131	-
Klebsiella_94	unsorted	dCTP diphosphatase activity	eggNOG-mapper	64313	64849	-
Klebsiella_95	replication	DNA primase activity	eggNOG-mapper	64857	65882	-
Klebsiella_96	unsorted	Prokaryotic dksA/traR C4-type zinc finger	eggNOG-mapper	65886	66146	-

Klebsiella_97	hypothetical	hypothetical protein	eggNOG-mapper	66202	66420	-
Klebsiella_98	assembly	Major capsid protein Gp23	eggNOG-mapper	66417	66905	-
Klebsiella_99	immune	methyltransferase activity	eggNOG-mapper	66911	67642	-
Klebsiella_100	hypothetical	hypothetical protein	eggNOG-mapper	67623	68015	-
Klebsiella_101	unsorted	Unknown	-	68012	68248	-
Klebsiella_102	hypothetical	hypothetical protein	eggNOG-mapper	68340	68594	-
Klebsiella_103	unsorted	Unknown	-	68674	68943	-
Klebsiella_104	replication	DNA helicase activity	eggNOG-mapper	70000	70423	-
Klebsiella_105	assembly	Head assembly gene product	eggNOG-mapper	70433	70753	-
Klebsiella_106	recombination	recA bacterial DNA recombination protein	eggNOG-mapper	70791	71948	-
Klebsiella_107	replication	nucleotide binding	eggNOG-mapper	72023	74722	-
Klebsiella_108	unsorted	Unknown	-	74769	75098	-
Klebsiella_109	hypothetical	hypothetical protein	eggNOG-mapper	75095	75382	-
Klebsiella_110	regulation	Bacteriophage translational regulator	eggNOG-mapper	75451	75813	-
Klebsiella_111	replication	Pfam:Phage_clamp_A	eggNOG-mapper	75817	76380	-
Klebsiella_112	unsorted	ATPase family associated with various cellular activities (AAA)	eggNOG-mapper	76383	77381	-
Klebsiella_113	replication assembly	gp45 sliding clamp, C terminal	eggNOG-mapper	77440	78105	-
Klebsiella_114	replication	Phage RNA polymerase binding, RpbA	eggNOG-mapper	78132	78401	-
Klebsiella_115	replication assembly	gp32 DNA binding protein like	eggNOG-mapper	78457	79428	-
Klebsiella_116	replication assembly	T4 gene Gp59 loader of gp41 DNA helicase	eggNOG-mapper	80000	80103	-
Klebsiella_117	unsorted	Pfam:Trans_coact	eggNOG-mapper	80100	80345	-
Klebsiella_118	regulation	Transcriptional regulator DsbA	eggNOG-mapper	80349	80612	-
Klebsiella_119	packaging	5'-3' exonuclease, N-terminal resolvase-like domain	eggNOG-mapper	80622	81557	-
Klebsiella_120	unsorted	sigma factor activity	eggNOG-mapper	81871	82389	+
Klebsiella_121	hypothetical	hypothetical protein	eggNOG-mapper	82386	82601	+

Klebsiella_122	unsorted	Protein of unknown function (DUF2654)	eggNOG-mapper	82594	82920	+
Klebsiella_123	unsorted	hydrolase activity	eggNOG-mapper	82957	84003	+
Klebsiella_124	hypothetical	hypothetical protein	eggNOG-mapper	83990	84277	+
Klebsiella_125	packaging	exonuclease activity	eggNOG-mapper	84261	85952	+
Klebsiella_126	replication	nucleic acid binding	eggNOG-mapper	85939	86658	-
Klebsiella_127	hypothetical	hypothetical protein	eggNOG-mapper	86715	86909	+
Klebsiella_128	replication	unfolded protein binding	eggNOG-mapper	86983	88692	+
Klebsiella_129	hypothetical	hypothetical protein	eggNOG-mapper	88752	89042	+
Klebsiella_130	hypothetical	hypothetical protein	eggNOG-mapper	89039	89269	+
Klebsiella_131	hypothetical	hypothetical protein	eggNOG-mapper	89266	89523	+
Klebsiella_132	replication	dihydrofolate reductase activity	eggNOG-mapper	90000	90098	+
Klebsiella_133	unsorted	Thymidylate synthase	eggNOG-mapper	90100	90975	+
Klebsiella_134	replication	Ribonucleotide reductase, barrel domain	eggNOG-mapper	91015	93267	+
Klebsiella_135	replication	Ribonucleotide reductase, small chain	eggNOG-mapper	93304	94479	+
Klebsiella_136	replication	hydroxymethylglutaryl-CoA reductase (NADPH) activity	eggNOG-mapper	94442	94771	+
Klebsiella_137	packaging	Endonuclease	Iterative search	94775	95140	+
Klebsiella_138	replication	RNA ligase	eggNOG-mapper	95121	96278	+
Klebsiella_139	lysis	Rz-like spanin	Iterative search	96287	96598	+
Klebsiella_140	lysis	Rz-like spanin	Iterative search	96595	96912	+
Klebsiella_141	unsorted	Unknown	-	96897	97070	+
Klebsiella_142	unsorted	AAA domain	eggNOG-mapper	97067	97951	+
Klebsiella_143	unsorted	Unknown	-	98004	98291	+
Klebsiella_144	unsorted	Unknown	-	98288	98539	+
Klebsiella_145	unsorted	Unknown	-	98532	98816	+
Klebsiella_146	replication	Cytidine and deoxycytidylate deaminase zinc-binding region	eggNOG-mapper	98758	99312	+

Klebsiella_147	unsorted	protein folding	eggNOG-mapper	99328	99669	+
Klebsiella_148	hypothetical	hypothetical protein	eggNOG-mapper	99680	99931	+
Klebsiella_149	hypothetical	hypothetical protein	eggNOG-mapper	100247	100528	+
Klebsiella_150	hypothetical	hypothetical protein	eggNOG-mapper	100538	100960	+
Klebsiella_151	hypothetical	hypothetical protein	eggNOG-mapper	101002	101568	+
Klebsiella_152	unsorted	Glycine radical	eggNOG-mapper	101579	101941	+
Klebsiella_153	hypothetical	hypothetical protein	eggNOG-mapper	101941	102210	+
Klebsiella_154	hypothetical	hypothetical protein	eggNOG-mapper	102336	102629	+
Klebsiella_155	unsorted	Unknown	-	102632	102871	+
Klebsiella_156	unsorted	Unknown	-	102856	103026	+
Klebsiella_157	hypothetical	hypothetical protein	eggNOG-mapper	103023	103175	+
Klebsiella_158	assembly	Bacteriophage protein GP30.3	eggNOG-mapper	103271	103711	+
Klebsiella_159	hypothetical	hypothetical protein	eggNOG-mapper	103708	103971	+
Klebsiella_160	hypothetical	hypothetical protein	eggNOG-mapper	103973	104194	+
Klebsiella_161	unsorted	hydrolase activity	eggNOG-mapper	104191	104805	+
Klebsiella_162	replication	DNA ligase (ATP) activity	eggNOG-mapper	104786	106315	+
Klebsiella_163	unsorted	Unknown	-	106390	106959	+
Klebsiella_164	hypothetical	hypothetical protein	eggNOG-mapper	107051	107323	+
Klebsiella_165	hypothetical	hypothetical protein	eggNOG-mapper	107385	107564	+
Klebsiella_166	unsorted	Unknown	-	107561	107752	+
Klebsiella_167	hypothetical	hypothetical protein	eggNOG-mapper	107749	108048	+
Klebsiella_168	hypothetical	hypothetical protein	eggNOG-mapper	108035	108412	+
Klebsiella_169	assembly infection	Baseplate structural protein, domain 1	eggNOG-mapper	108409	109518	-
Klebsiella_170	infection	Baseplate hub distal subunit	eggNOG-mapper	110000	110163	+
Klebsiella_171	infection	baseplate hub	Iterative search	110160	111941	+
Klebsiella_172	infection	baseplate tail tube cap	Iterative search	111952	113025	+
Klebsiella_173	assembly infection	T4-like virus tail tube protein gp19	eggNOG-mapper	113035	113898	+

Klebsiella_174	infection	T4 bacteriophage base plate protein	eggNOG-mapper	113927	114670	+
Klebsiella_175	infection	T4 bacteriophage base plate protein	eggNOG-mapper	114667	115221	+
Klebsiella_176	lysis	Gene 25-like lysozyme	eggNOG-mapper	115222	115614	+
Klebsiella_177	hypothetical	hypothetical protein	eggNOG-mapper	115649	116104	-
Klebsiella_178	hypothetical	hypothetical protein	eggNOG-mapper	116112	116336	-
Klebsiella_179	unsorted	Unknown	-	116338	116622	-
Klebsiella_180	hypothetical	hypothetical protein	eggNOG-mapper	116694	116978	-
Klebsiella_181	assembly infection	head-tail adaptor	Iterative search	116994	117464	-
Klebsiella_182	unsorted	Unknown	-	117461	117619	-
Klebsiella_183	hypothetical	hypothetical protein	eggNOG-mapper	117616	118266	-
Klebsiella_184	unsorted	von Willebrand factor type A domain	eggNOG-mapper	120000	120521	-
Klebsiella_185	hypothetical	hypothetical protein	eggNOG-mapper	120529	120849	-
Klebsiella_186	hypothetical	hypothetical protein	eggNOG-mapper	120849	121364	-
Klebsiella_187	unsorted	Unknown	-	121361	121603	-
Klebsiella_188	hypothetical	hypothetical protein	eggNOG-mapper	121590	121877	-
Klebsiella_189	unsorted	Unknown	-	121877	122044	-
Klebsiella_190	unsorted	Unknown	-	122084	122362	-
Klebsiella_191	regulation	C-5 cytosine-specific DNA methylase	eggNOG-mapper	122421	123332	-
Klebsiella_192	hypothetical	hypothetical protein	eggNOG-mapper	123329	123586	-
Klebsiella_193	unsorted	Unknown	-	123586	123831	-
Klebsiella_194	immune	Predicted nucleotidyltransferase	eggNOG-mapper	123828	124835	-
Klebsiella_195	hypothetical	hypothetical protein	eggNOG-mapper	124835	125212	-
Klebsiella_196	hypothetical	hypothetical protein	eggNOG-mapper	125209	125436	-
Klebsiella_197	unsorted	Unknown	-	125433	126038	-
Klebsiella_198	hypothetical	hypothetical protein	eggNOG-mapper	126041	126313	-
Klebsiella_199	hypothetical	hypothetical protein	eggNOG-mapper	126310	126504	-
Klebsiella_200	unsorted	NUDIX domain	eggNOG-mapper	126678	127739	-
Klebsiella_201	unsorted	SPFH domain / Band 7 family	eggNOG-mapper	127916	128830	-

Klebsiella_202	regulation	starvation-inducible transcriptional regulator	Iterative search	128902	129324	-
Klebsiella_203	lysis regulation	lysis inhibition	Iterative search	129296	129577	-
Klebsiella_204	hypothetical	hypothetical protein	eggNOG-mapper	130000	130076	-
Klebsiella_205	unsorted	thymidine kinase activity	eggNOG-mapper	130093	130701	-
Klebsiella_206	unsorted	Unknown	-	130884	131099	-
Klebsiella_207	unsorted	Unknown	-	131092	131379	-
Klebsiella_208	lysis	cysteine-type peptidase activity	eggNOG-mapper	131382	131855	-
Klebsiella_209	unsorted	Unknown	-	132046	132162	-
Klebsiella_210	tRNA	valyl tRNA synthetase modifier	Iterative search	132162	132485	-
Klebsiella_211	hypothetical	hypothetical protein	eggNOG-mapper	132495	133085	-
Klebsiella_212	unsorted	Pfam:DUF5484	eggNOG-mapper	133082	133228	-
Klebsiella_213	hypothetical	hypothetical protein	eggNOG-mapper	133239	133634	-
Klebsiella_214	lysis	lytic transglycosylase activity	eggNOG-mapper	133702	134343	-
Klebsiella_215	hypothetical	hypothetical protein	eggNOG-mapper	134408	134962	-
Klebsiella_216	hypothetical	hypothetical protein	eggNOG-mapper	134974	135171	-
Klebsiella_217	unsorted	PhoH-like protein	eggNOG-mapper	135208	136551	-
Klebsiella_218	hypothetical	hypothetical protein	eggNOG-mapper	136607	137065	-
Klebsiella_219	lysis	D-alanyl-D-alanine carboxypeptidase	eggNOG-mapper	137097	137492	-
Klebsiella_220	hypothetical	hypothetical protein	eggNOG-mapper	137560	137961	-
Klebsiella_221	hypothetical	hypothetical protein	eggNOG-mapper	138016	138339	-
Klebsiella_222	hypothetical	hypothetical protein	eggNOG-mapper	138379	138642	-
Klebsiella_223	hypothetical	hypothetical protein	eggNOG-mapper	138712	138972	-
Klebsiella_224	hypothetical	hypothetical protein	eggNOG-mapper	138974	139405	-
Klebsiella_225	unsorted	Unknown	-	139416	139589	-
Klebsiella_226	hypothetical	hypothetical protein	eggNOG-mapper	139586	139933	-
Klebsiella_227	hypothetical	hypothetical protein	eggNOG-mapper	140000	140279	-
Klebsiella_228	unsorted	Unknown	-	140263	140565	-

Klebsiella_229	replication	Iron-sulfur cluster-binding domain	eggNOG-mapper	140562	141764	-
Klebsiella_230	unsorted	Unknown	-	141718	141924	-
Klebsiella_231	hypothetical	hypothetical protein	eggNOG-mapper	141921	142184	-
Klebsiella_232	hypothetical	hypothetical protein	eggNOG-mapper	142250	142420	-
Klebsiella_233	hypothetical	hypothetical protein	eggNOG-mapper	142417	142614	-
Klebsiella_234	unsorted	Unknown	-	142611	142760	-
Klebsiella_235	hypothetical	hypothetical protein	eggNOG-mapper	142757	143008	-
Klebsiella_236	hypothetical	hypothetical protein	eggNOG-mapper	143080	143358	-
Klebsiella_237	assembly	head protein	Iterative search	143427	143759	-
Klebsiella_238	unsorted	Unknown	-	143838	144095	-
Klebsiella_239	hypothetical	hypothetical protein	eggNOG-mapper	144095	144670	-
Klebsiella_240	unsorted	Unknown	-	144678	145076	-
Klebsiella_241	unsorted	Unknown	-	145251	145451	-
Klebsiella_242	unsorted	Unknown	-	145528	146442	-
Klebsiella_243	unsorted	Protein of unknown function DUF262	eggNOG-mapper	146439	146966	-
Klebsiella_244	hypothetical	hypothetical protein	eggNOG-mapper	146966	147289	-
Klebsiella_245	hypothetical	hypothetical protein	eggNOG-mapper	147318	147575	-
Klebsiella_246	hypothetical	hypothetical protein	eggNOG-mapper	147577	147960	-
Klebsiella_247	unsorted	NUDIX domain	eggNOG-mapper	147971	148378	-
Klebsiella_248	unsorted	Unknown	-	148379	148624	-
Klebsiella_249	unsorted	Unknown	-	148621	148872	-
Klebsiella_250	hypothetical	hypothetical protein	eggNOG-mapper	148865	149158	-
Klebsiella_251	hypothetical	hypothetical protein	eggNOG-mapper	149155	149490	-
Klebsiella_252	hypothetical	hypothetical protein	eggNOG-mapper	149501	149641	-
Klebsiella_253	hypothetical	hypothetical protein	eggNOG-mapper	150000	150476	-
Klebsiella_254	replication	RNA ligase	Iterative search	150484	151107	-
Klebsiella_255	infection	tail fiber chaperone	Iterative search	151159	151431	-
Klebsiella_256	unsorted	kinase activity	eggNOG-mapper	151434	152129	-
Klebsiella_257	assembly infection	T4-like virus tail tube protein gp19	eggNOG-mapper	152129	152659	-
Klebsiella_258	hypothetical	hypothetical protein	eggNOG-mapper	153028	153816	+

Klebsiella_259	unsorted	DNA end protector	Iterative search	153853	154707	-
Klebsiella_260	assembly	head closure	Iterative search	154723	155190	-
Klebsiella_261	infection	Base plate wedge protein 53	eggNOG-mapper	155241	155795	+
Klebsiella_262	regulation assembly	cell wall macromolecule catabolic process	eggNOG-mapper	155792	157561	+
Klebsiella_263	hypothetical	hypothetical protein	eggNOG-mapper	157563	159629	+
Klebsiella_264	assembly infection	virus tail assembly	eggNOG-mapper	160000	161586	+
Klebsiella_265	assembly infection	virus tail assembly	eggNOG-mapper	161667	164753	+
Klebsiella_266	assembly	Bacteriophage T4, Gp8	eggNOG-mapper	164754	165746	+
Klebsiella_267	assembly	Bacteriophage T4 gp9/10-like protein	eggNOG-mapper	165756	166619	+
Klebsiella_268	assembly	Bacteriophage T4 gp9/10-like protein	eggNOG-mapper	166616	168433	+
Klebsiella_269	assembly infection	GP11 baseplate wedge protein	eggNOG-mapper	168433	169101	+
Klebsiella_270	infection	virus tail, fiber	eggNOG-mapper	170000	170496	+
Klebsiella_271	infection	Virion	eggNOG-mapper	170507	172258	+
Klebsiella_272	infection	Virion	eggNOG-mapper	172297	173223	+
Klebsiella_273	assembly	Virus neck protein	eggNOG-mapper	173233	173979	+
Klebsiella_274	assembly	Pfam:T4-gp15_tss	eggNOG-mapper	174059	174589	+

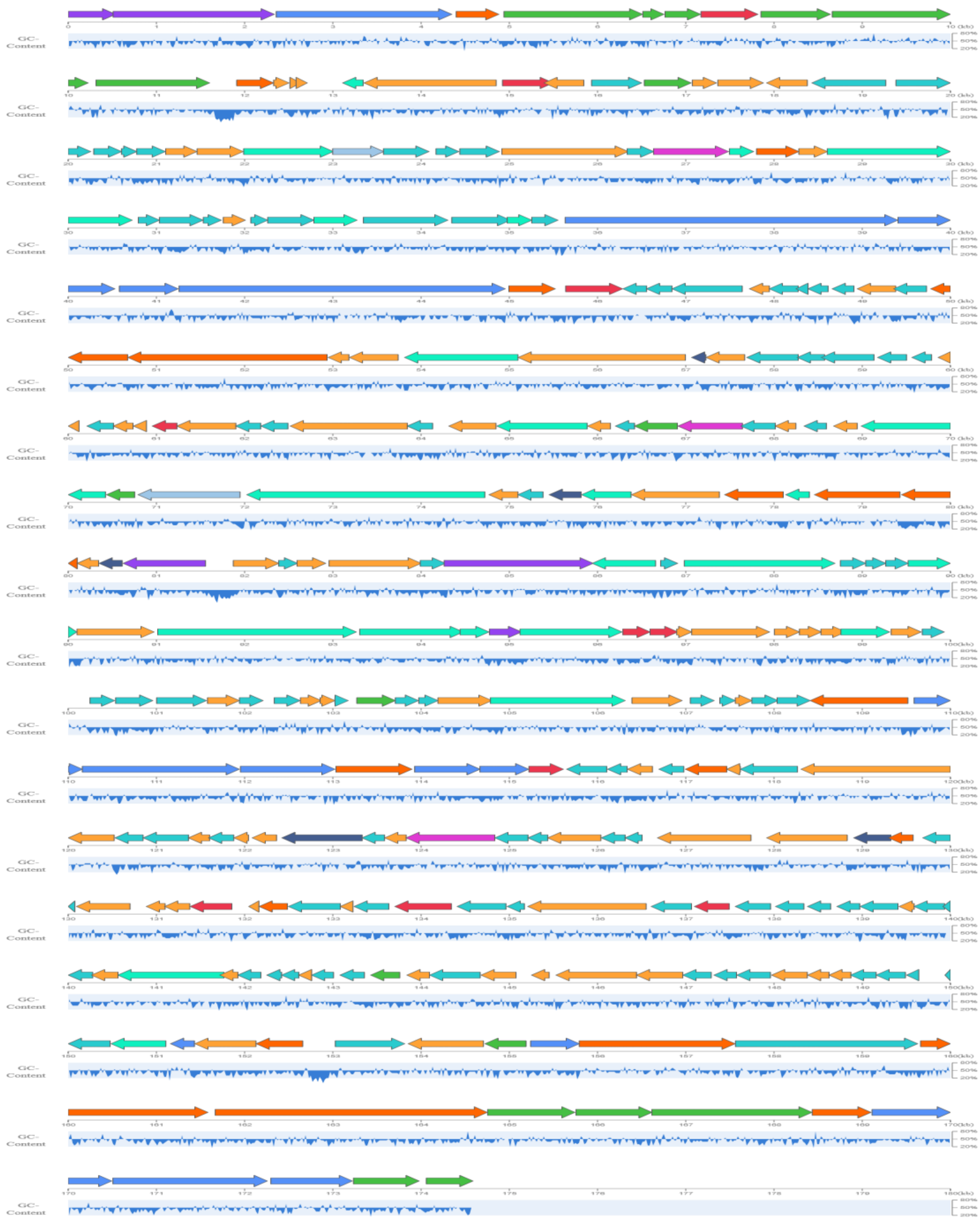

Supplementary Figure S1: 274 ORFs are shown in the genetic map of the *Klebsiella pneumoniae phage* KP Ø1 phage using PhageScope with TaskID: 41733. The function of each ORF is indicated by different colors:

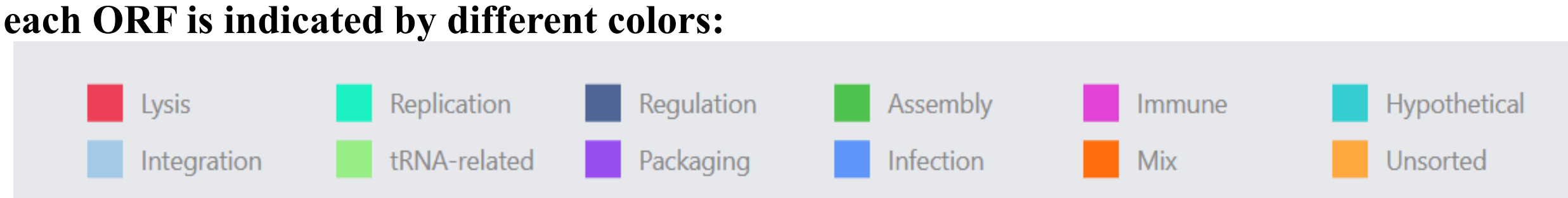

Supplement: Supplementary file 1 [file 41598_2026_67695_MOESM1_ESM.pdf]
